# Evaluating the bio-economic performance of a *Callo de hacha (Atrina maura*, *Atrina tuberculosa & Pinna rugosa)* fishery restoration plan in La Paz, Mexico

**DOI:** 10.1371/journal.pone.0209431

**Published:** 2018-12-20

**Authors:** Juliano Palacios-Abrantes, Juliana Herrera-Correal, Salvador Rodríguez, Jacy Brunkow, Renato Molina

**Affiliations:** 1 Bren School of Environmental Science & Management, University of California, Santa Barbara, United States of America; 2 Rosenstiel School of Marine and Atmospheric Science, University of Miami, Miami, United States of America; 3 Department of Economics, University of Miami, Miami, United States of America; University of New Haven, UNITED STATES

## Abstract

Small-scale fisheries are large contributors to regional economies and livelihoods in coastal communities of Latin America. While Mexico is one of the cases where small-scale fisheries play an important role, overfishing and poor management strategies have led to the collapse of many of its fisheries. The *callo de hacha* scallop fishery of the *Ensenada* de La Paz in Baja California Sur is an example of such a fishery which, after years of mismanagement, was closed by the Mexican authorities in 2009. The present study evaluated the recovery efforts in the cove and the potential outcomes of a collaboration between a non-governmental organization and a fishing community working towards the restoration of this pen-shell fishery. After more than four years of closure and active monitoring of the recovering process, the *callo de hacha* population has shown a significant population recovery, with potential solvency for reopening fishing activities. Four scenarios of uncertainty are evaluated with two of them providing positive net present values from reopening the fishery. We also document the involvement of a non-governmental organization with a fishing community, which created social capital and, in our opinion, was essential for a successful restoration. Having an actively involved community helped raise funds for the fishing closure so fishers were able to comply with Mexican legislation; it also fostered community building and self-organization that will be crucial to maintaining the sustainability of the fishery.

## Introduction

Small-scale fisheries in Latin America have been increasingly recognized for their contribution to regional economies as well as for their importance to the livelihoods of coastal communities [1]. Unfortunately, the inherent traits of these fisheries present challenges for proper management [2]. This is especially the case for Mexico, one of Latin America’s largest seafood producing countries, where 97% of all fishing vessels can be characterized as small-scale or artisanal [2], and where there has been clear evidence of widespread overfishing and poor management across the sector [3,4]. Some examples of this include the *chocolata* scallop (*Megapitaria squalida*) [5]; the blue abalone (*Haliotis fulgens*) and the yellow abalone (*Haliotis corrugata*) along the Baja California Peninsula on the Pacific coast [6]; and pelagic fishes like mackerel *(Scomberomorus maculatus)* and red snapper *(Lutjanus campechanus)*, on the Atlantic coast [3]. In the face of this pressing issue, restoring depleted stocks becomes a priority so that communities that depend on them can reap the benefits of sustainable management and ensure their future livelihoods [4,7,8].

This study examines a community-driven effort to recover a small-scale fishery in the cove (referred to as the *Ensenada)* of La Paz, located in Baja California Sur, Mexico. The fishery is locally known as *callo de hacha* even though it includes three species of pen-shell scallops: *Atrina maura*, *Atrina tuberculosa and Pinna rugosa*. These scallops are among the highest valued small-scale seafood products harvested in the region [9]. Although, historically, a single kilogram has reached a dock price of US$23 per kg in La Paz [10], fishers personally reported to the authors a current price (April/2018) of over US$ 21 per kg. This reported price is 50% higher than that for other comparable mollusk in the Mexican seafood market [10].

Mexican fisheries management falls under the umbrella of several sub-agencies within the Ministry of Agriculture, Livestock, Rural Development, Fisheries and Food *(“Secretaría de Agricultura*, *Ganadería*, *Desarrollo Rural*, *Pesca y Alimentación”; SAGARPA)*. The main guiding regulation is dictated by the “*Ley General de Pesca y Acuacultura Sustentables (LGPAS)”* [11], which establishes the obligations and guidelines for most fisheries in the country through the “*Carta Nacional Pesquera*” [12]. By law, the Fisheries Institute (*Instituto Nacional de Pesca;* INAPESCA) is in charge of recommending fishing quotas to the Fisheries Council (*Consejo Nacional de Pesca;* CONAPESCA), which is then responsible for implementing the management actions necessary to comply with that recommendation. The monitoring and enforcement of activities are then the responsibility of both CONAPESCA and the Navy (*Secretaría de Marina*) [11]. Despite these requirements, fisheries like the *callo de hacha* lack a proper management plan or official standard [12] resulting in most small scale fisheries in the country operate under de facto open access [13,14]. In the *callo de hacha* fishery of the cove of La Paz, these practices led to its collapse in 2008 [15,16]. According to stakeholders, years of mismanagement, corruption, and poor enforcement in the fishery had led to such a degree of overexploitation that it had to be officially closed [15,16]. As a moratorium on fishing went into effect, fishers were faced with the loss of an important source of income. This created a strong incentive not to respect the moratorium and to continued fishing for the resource both in the cove and in other locations [15,16].

This situation led to a local effort to recover the fishery and, with it, the stability of the fishing community. A local NGO, *Noroeste Sustentable* (NOS), partnered with *El Mangle* fishing community of the cove to launch a joint restoration plan. The initiative sought to increase biomass up to that necessary for a sustainable commercial harvest while, at the same time, to help the community gain property rights over the resource and incentivize a sustainable stewardship of the resource [17,18]. Gaining property rights over the resource was essential for the community if they were to benefit from the restoration plan and ensure the sustainable fishery [4]. NOS was able to fund fishers to engage in several activities related to the recovery plan that resulted in more than four years of active enforcing and monitoring of the *callo de hacha* biomass. The recovery plan was careful to consider not only the biological conditions of the stock, but also the economic and legal frameworks governing the fishery, as well as the social dynamics of the fishing community; all previously identified as important components of fisheries restoration [16,19–21].

Social capital, defined as the rules and community networks that facilitate co-operation, seems to be key in developing sustainable co-management of small-scale fisheries in Latin America [22]. The recovery program successfully incorporated this notion and worked to build social capital by engaging with the community to co-develop their social, economic, and environmental goals. The program focused on promoting self-organization and self-enforcement, as well as on organizing information campaigns that were used to disseminate sustainable fishing practices. Workshops led by NOS also provided a space to discuss the community’s organization, mend internal issues, and determine management strategies and fishing policies. These activities gave the fishers a sense of ownership over the resource, allowed them to set their own internal rules for enforcement, and promoted sustainable harvest methods. Finally, the process also helped overcome differences within the community and resulted in fishers forming a cooperative collective. The collective, or organization (“*Organización de Pescadores Rescatando la Ensenada*”), was key to controlling illegal harvest as well as to securing property rights over a resource that eventually recovered. This transition led the fishery, at least from the fishers’ perspective, from de facto open access to a property-right based system [17,18].

While many examples of successful stock recoveries have been documented for a wide array of species, including king mackerel (*Scomberomorus cavalla)*, stiped bass (*Marone saxatilis)*, and deep-sea scallops (*Placopecten magellanicus)* [21], there is very little analysis as to how Mexican fisheries have performed [3]. This statement is also true for the *callo de hacha* in La Paz. In this context, and given the potential for reopening this high-value fishery, the objective of this study was to evaluate the performance of the restoration plan in terms of biomass recovery and the likeness of its economic solvency. Our analysis was based on biological data collected since the fishery closed as well as an appraisal of the best available economic variables for its operation. Our results suggest that, after four years (2011–2014), the resource substantially recovered, potentially making the fishery profitable with significant benefits for the community. Nevertheless, high uncertainty in the biological samples hinders economic analysis, concealing the true potential benefits of the *callo de hacha* fishery. Our results suggest that, in some cases, the fishery could be profitable; however, our most pessimistic scenarios provide a warning against overly positive expectations.

## Materials and methods

To assess the success of the current restoration plan in economic terms, both biomass and the economic value of the fishery were estimated. Methods are then divided into three sections: biomass estimation, economic analysis, and data sources.

### Biomass estimation

Total biomass of the fishery was estimated using size structure and mean abundance [23]. First, individual weight was calculated from individual size using an *allometric* individual growth equation [24]:

$$W=a(L^{b})$$ (1)

where *a* is a constant, *L* is the organism’s size and *b* is the species’ intrinsic growing rate. Values of *a* and *b* were obtained from Serrano-Guzmán (2004) [24]. Second, mean weight ($\bar{W}$) was estimated using the following equation [23]:

$$\bar{W}=\sum_{i=1}^{n}\frac{X_{i}}{n_{i}}$$ (2)

where *Xi* is the weight of individuals on quadrat *i*, and *n* is the total sampled area for each quadrat. For the purpose of clarity, time indexes are omitted. Average weight was then used to calculate the average biomass (*B*) by multiplying *W* by the total area encompassed by the resource. Mean weight and biomass variances were then estimated. Finally, both upper and lower 95% confidence intervals (CI) were generated using the method described in DeLong *et al* (1988) [25]. Due to government-imposed size limits [12], the harvestable biomass was estimated from those individuals larger than 14 cm in length. Finally, we also took into account the fact that *callo de hacha* is not sold as the whole individual, but rather as just the edible portion of the animal known as “muscle” (anterior adductor). Thus, estimations of harvestable biomass were subsequently converted into edible biomass through a 25 percent individual to muscle ratio [26].

A caveat in this analysis is the unfeasibility of projecting the population dynamics into the future due to data restrictions and the number of observations available. An initial attempt to establish a relationship between spawning biomass and individual recruits had to be discarded due to insufficient data-points (S1 Fig). In addition, there was limited substantiated biological knowledge of the stock to determine if the population was self-maintained, or larvae was supplied from external sources. To tackle this limitation, while still providing insights on the economic feasibility of the fishery, the population was assumed to be in equilibrium in the last sample due to the low degree of variation with respect to the previous year. Projections were then done assuming the that the stock will be in equilibrium at a fixed fishing mortality. For each year, harvestable biomass available (*H*) was randomly projected assuming a normal distribution with a noise component as follows:

$$H(\pi |\mu ,\sigma^{2})=\frac{1}{\sqrt{2\pi }\sigma^{2}}e^{-\frac{\left(R-\mu^{2}\right)}{2\sigma^{2}}}$$ (3)

where *π* is a normal random variable, (*μ*) is the mean equilibrium biomass over 14 cm, and (σ) is the standard deviation. For all years, fishing mortality was set to 20% of *H*, as recommended by the Mexican fishing law [12] and applied by similar fisheries [23]. By including a random factor in the estimations, this analysis took into consideration that the stock may experience timely variations that influence the availability of harvestable biomass each year. Given all of these assumptions for the analysis (S1 Table), the estimates presented are likely to be conservative. The logic behind this claim relies on the fact that if the stock is still on its way to recover to a higher level of equilibrium biomass, then we are in fact using a lower point of production that could actually be sustained in the future.

### Economic analysis

The economic model was developed in order to estimate profitability of the callo de hacha fishery. The model is available in a friendly format (Excel) and can be adapted to other fisheries (S2 File). The analysis considers different steps along the fishing process (i.e., harvestable biomass, selling price, and fishing costs) and it works as a scenario simulator. To calculate the total profits generated, catch (*C*) was estimated in every simulation from a normal-distribution random value as follows:

C=(H*F)*CF (4)

where *F* is fishing mortality and *CF* is conversion factor of individual-muscle. Total fishing costs (*Tc*) were estimated based on fixed and variable costs of the fishery. The fixed cost was divided into operational costs and vessel-specific costs, while the variable cost was divided in cost per trip and cost per unit of catch. This structure is justified because the callo de hacha fishery has yearly fixed costs associated with vessel operation and access to the fishery, and variable costs associated with fishing effort and product handling. For example, each fisher must pay a yearly fishing and vessel permit. For each vessel used (unit of effort), the fishers must cover gas and oil. Once the fishing operation takes place, costs like ice bags and fuel costs of delivery depend on the amount of harvest (unit of catch). Operational fishing cost (*OFC*) was calculated as:

$$OFC=\sum_{j=1}^{n}Yc$$ (5)

where *Yc* equals yearly costs related to the operation of the fishery, and *j* equals the different sources of operational cost. For the purpose of clarity, the time index has also been omitted. Vessel fixed cost (*VFC*) was estimated by:

$$VFC=(\sum_{j=1}^{n}Bc)*Bu$$ (6)

where *Bc* represents yearly fixed costs related only to boat availability and *Bu* represents the relative fraction of the total boat’s capacity used to target callo de hacha. Finally, variable cost (*VC*) was computed as:

Vc=−(E*Ce)+(C*Cc) (7)

where *E* is the effort (number of trips needed to harvest *C*), *Ce* is cost per unit of effort, defined by the sum of the costs per fishing trip, and *Cc* is cost per unit of catch. Total fishing cost (*Tc*) is given by the sum of *OFC*, *VFC* and *Vc*.

Fishing revenue (*R*) was estimated by multiplying *C* by the callo-muscle ratio times the selling price. The Net Present Value (NPV) was discounted and projected over a 10-year period as follows:

$$NPV=\sum_{y=0}^{y=10}\frac{R-(Tc+Tax)}{{\left(1+r\right)}^{y}}$$ (8)

Where *Tax* is tax expenses, *y* represents the current year, and *r* is the discount rate. The discount rate was assumed to be 10%. Additional discount rates (20, 30, 40, and 50%) were used to test the sensitivity of the model to this assumption.

We conducted a Monte Carlo simulation with a total of 1000 runs per scenario. For each simulation year, the model randomly determined the harvest biomass available from the mean (± s.d) equilibrium biomass (Eqs 3 and 4). All other variables remained constant throughout the simulations.

### Data sources

The analyses in this paper were conducted using the biologic data collected by the fishers for the three callo de hacha species (*Atrina maura*, *A*. *tuberculosa*, and *Pinna rugosa*) that constitute the fishery in the *Ensenada* of La Paz. Data was gathered by fishers with support from NOS through an annual monitoring campaign from 2011 until 2014 (Raw data in S1 Data). Given that these three species share similar biological characteristics, the entire sampled population was treated as one fishable stock [27]. This assumption is feasible because the analysis does not model biological dynamics of the species. Despite the loss of specificity with this assumption, the simplification allowed for a broader sense of the profitability of the fishery as whole.

The total habitable area of the resource in the cove of La Paz is 1330 ha., however, biological sampling was limited to 176 ha, divided into quadrats of one ha. In each quadrat, fishers measured up to 114 individuals and then counted the rest (without measuring them). A density higher than 114 individuals per quadrat was only found in the samples from 2013 and 2014 (Table 1).

**Table 1:** Number of quadrats monitored per year.

Description	Year			
	2011	2012	2013	2014
**Total number of quadrats sampled**	116	121	130	140
**% of quadrats with measured individuals**	100	100	100	100
**% of quadrats with counted individuals**	0	0	4	12

The table shows the percentage of quadrats where individuals were measured and counted separately. Percentages do not add up to a 100, as quadrats that were counted were also measured. Note that the area used to estimate the biomass in each sample was aligned with the number of quadrats monitored.

The raw data presented several inconsistencies through the monitoring years that we considered when performing this analysis. First, to estimate the size of the individuals that were counted but not measured, we used a proportion of the measured gross weight (Eq 1). We assumed that the sample size structure adequately represented the overall size structure in each quadrat. For example, if 10 percent of the measured individuals had 18 cm in length and thus weighted 100 g each, then 10 percent of the entire quadrat individuals counted had the same length, and thus the same individual weight. Second, 16 quadrats—four in 2013 and 12 in 2014—presented consistently higher biomass than all other quadrats in the sample for those years. To deal with this pattern, we assumed that there were two subpopulations in the stock, one where quadrats presented high-densities, and another where quadrats had low-densities. Third, and final, the sampling area was inconsistent throughout the years due to different methods used (i.e., 5 m transects vs 50 m transects per quadrat) and a different number of quadrats sampled. Therefore, to determine the total biomass we extrapolate from the sample to the entire quadrat and to the cove, taking into consideration as part of the method the different areas that were sampled.

**Fig 1:**
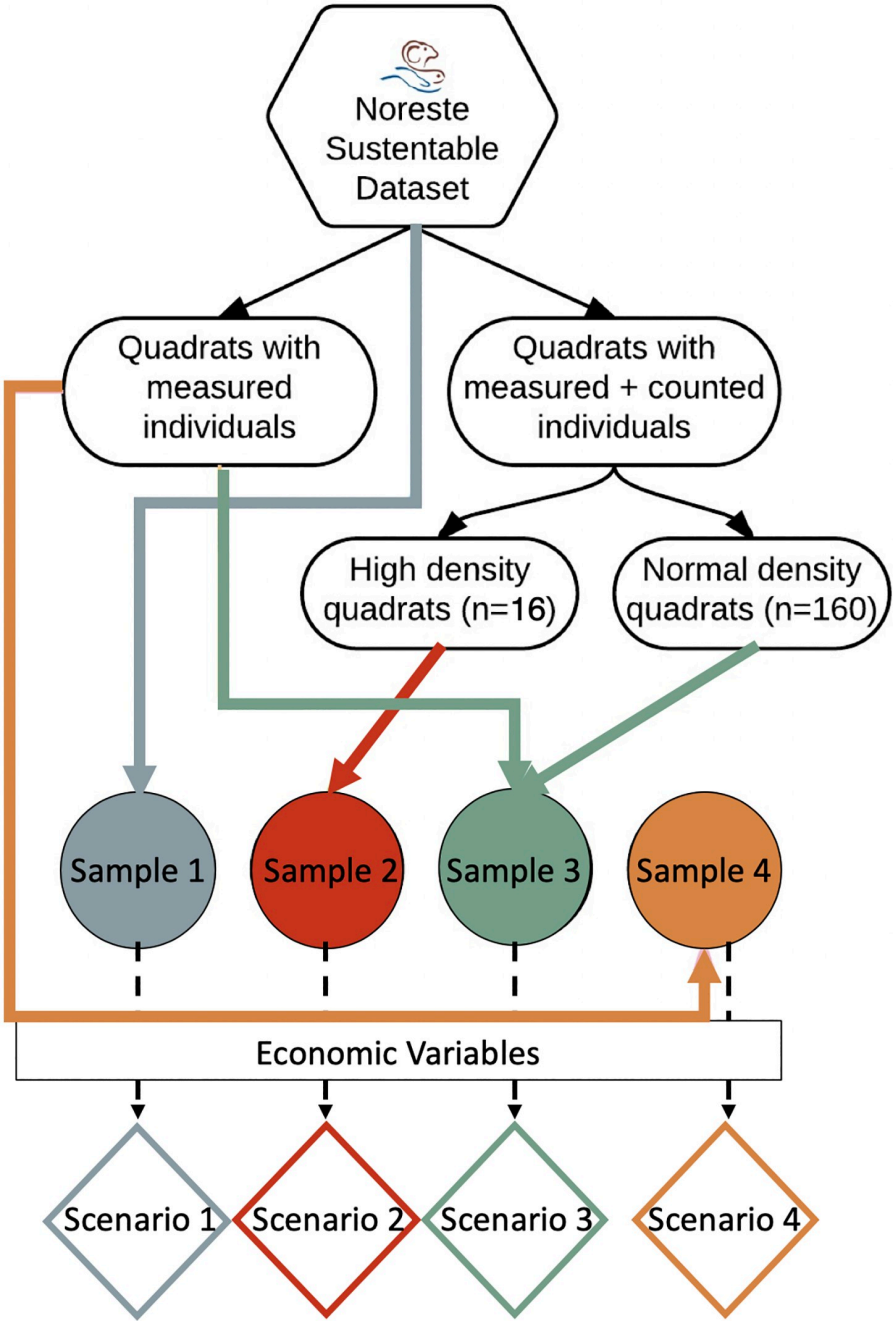
Method followed to determine the four biomass samples. Sample 1) considers counted and measured individuals in all quadrats; sample 2) only considers quadrats with the highest abundance during 2013 and 2014; sample 3) considers only low abundance quadrats with counted and measured individuals, and sample 4) considers the quadrats that only measured individuals. Each sample was used to estimate the status of the fishery and represent a different harvestable scenario for the economic analysis.

To capture the uncertainty associated with the sampling methods, as well as the overall abundance reported by its results, we subdivide the data into four samples (Fig 1). Each one of these samples represent a different potential biomass estimation within the cove, which we then used to estimate the random parameters used in the economic analysis (*μ* ± *σ* Eq 3). Sample 1 accounts for the whole dataset, including counted and measured individuals as well as all quadrats. Sample 2 includes only the quadrats with high biomass during the last two years of monitoring. Sample 3 excludes the quadrats from Sample 2 and accounts for both measured and counted individuals. Finally, Sample 4 includes only those quadrats where individuals were measured, but not counted. Samples 1, 3 and 4 were extrapolated to 1330 ha, while Sample 2 was extrapolated to 4 ha in 2013 and 12 ha 2014, according to the amount of sampled quadrats with high biomass.

Based on the four samples, we developed four economic scenarios to establish the profitability of the *callo de hacha* fishery (Fig 1). Each scenario differs from the other solely by equilibrium biomass. Thus, all economic variables and management assumptions remained fixed across the analysis. Fisheries and economic parameters directly related to the *callo de hacha* fishery were obtained from informal interviews with fishers, managers and NOS personnel. Grey literature was also used when no scientific literature or legal documents were available (Table 2). For all values, dollar exchange rate was taken from *Banamex* Bank in April 26, 2016 (US$ 1 = MX $16.82). All graphic results were done using *R*-*Studio* software with the *ggplot2* [28] and *wesanderson* [29] packages.

**Table 2:** Biological, economic and fishery parameters to run the model for each scenario.

Parameters	Value	Source
Mean (*s*.*d*.) biomass Sample 1	35 *(16)* tons	Fishers database
Mean (*s*.*d*.) biomass Sample 2	30 *(14*.*8)* tons	Fishers database
Mean (*s*.*d*.) biomass Sample 3	4.5 *(1*.*6)* tons	Fishers database
Mean (*s*.*d*.) biomass Sample 4	3.4 (*1*.*1*) tons	Fishers database
Conversion factor	0.25 kg of muscle/kg	Interview with manager[26]
Initial Investment	MX$ 390, 000.00	NOS
Ex-vessel price per kilo	MX$ 200.00	Interview with fishers
Fixed cost^1^	MX$ 127,752.00	Interview with fishers
Cost per unit of effort^2^	MX$ 220.00	Interview with fishers
Cost per unit of catch^3^	MX$ 0.80	Interview with fishers
Depreciation	10%	Model Assumption
Tax	15%	Model Assumption
Discount rate^4^	10%	Model Assumption
Fishing mortality	20%	Literature [12,23]
Catch per unit of effort (CPUE)	25 kg/unit of effort	Interview with fishers

^*1*^*Fixed cost* includes fishing permit, boating permit, gear maintenance, other expenses, boat maintenance, engine maintenance, enforcement; weighted by the boat percentage used by the fishery.

^2^*Cost per unit of effort* includes gasoline, oil and fisher salary.

^3^*Cost per unit of catch* includes: gasoline, shipment cost, ice bag.

^4^Discount rate was ranged from 5–15%.

## Results

### Biological results

According to our analysis, biomass significantly increased for all four biological samples as a result of the closure in the cove (Table 3). In particular, for samples 1 and 2, total and harvestable biomass increased threefold from 2013 to 2014. As expected, our estimations yielded the highest value of total biomass for Sample 1. For samples 3 and 4, total biomass slightly increased over time, however, values did not change as dramatically as the first two samples would suggest (Table 3). Samples 1 and 2 showed the same trend until 2013, when the fishery started to show signs of recovery, as biomass reached 4.44 tons for Sample 3 and 5.98 tons for Sample 4, almost double the values in 2011. Overall, Sample 4 showed the lowest biomass increase over time.

**Table 3:** Estimates of total and harvestable and edible biomass (tons) in the *Ensenada*.

		2011	2012	2013	2014
**Sample 1** mean(±*s*.*d*.)	**Total**	—	—	14.46 *(3*.*78)*	45.56 *(26*.*22)*
	**Harvestable**	—	—	12.39 *(5*.*36)*	35.02 *(24*.*81)*
	**Edible**	—	—	3.09	8.76
**Sample 2** mean(±*s*.*d*.)	**Total**	—	—	10.88 (*5*.*43)*	36.58 (*29*.*12)*
	**Harvestable**	—	—	9.54 (*5*.*3)*	30.49 (*24*.*71)*
	**Edible**	—	—	2.38	7.62
**Sample 3** mean(±s.d.)	**Total**	2.76 *(0*.*63)*	2 (*0*.*39)*	3.58 (*1*.*01)*	4.44 (*1*.*63)*
	**Harvestable**	1.21 (*0*.*26)*	1.26 (*0*.*27)*	2.85 (0.82)	3.48 (*1*.*5)*
	**Edible**	0.30	0.31	0.71	0.87
**Sample 4** mean(±s.d.)	**Total**	2.76 *(0*.*63)*	2 *(0*.*4)*	3.58 (*1*.*01)*	5.98 (*2*.*44)*
	**Harvestable**	1.21 (*0*.*57)*	1.26 (*0*.*27*)	2.85 (0.82)	4.54 (*0*.*95)*
	**Edible**	0.30	0.31	0.71	1.13

No differences were found between Samples 3 and 4 until 2014.

### Economic results

**Fig 2:**
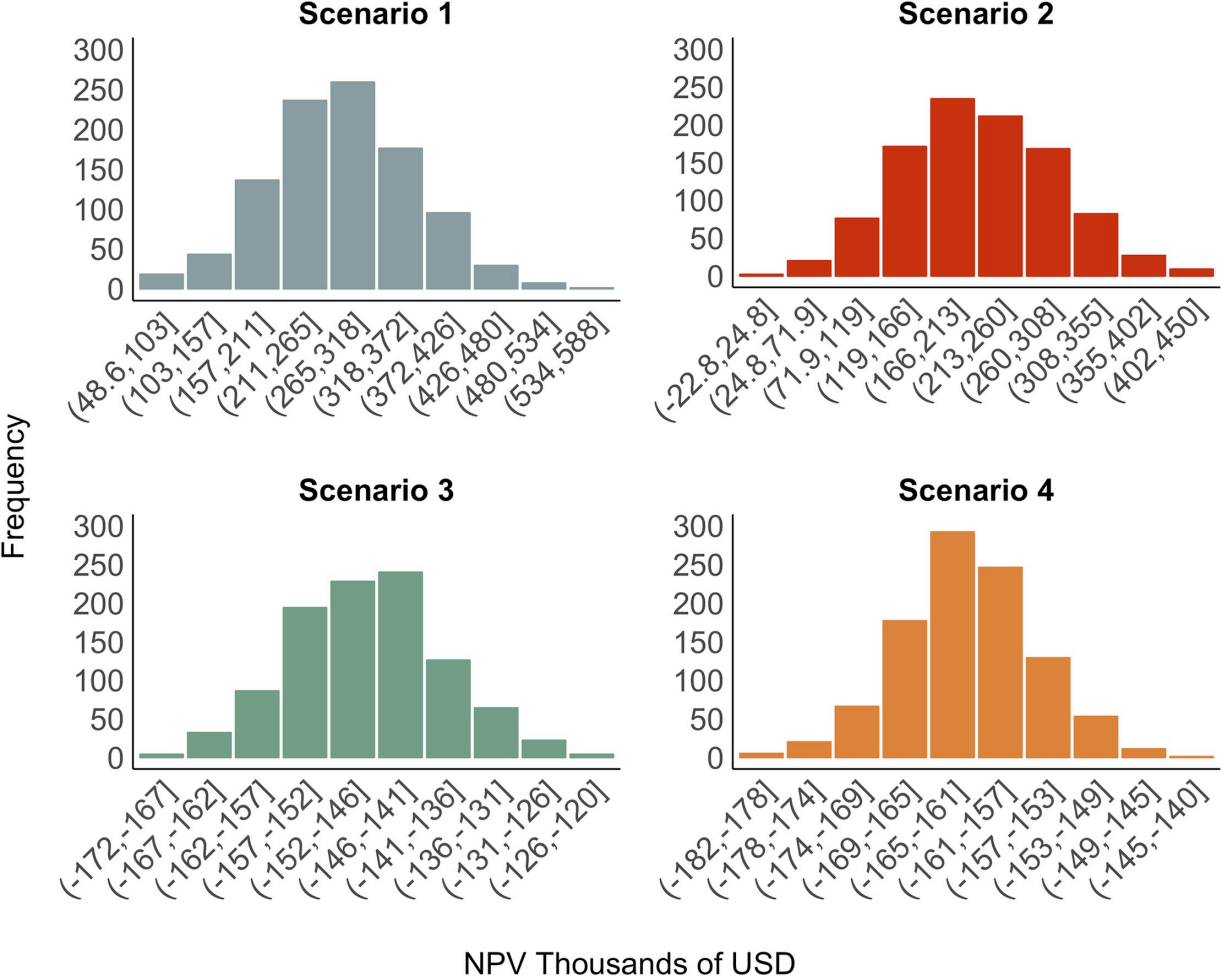
Net present value simulations for each *callo de hacha* scenario. 1000 simulations were carried for each scenario.

After performing the Monte Carlo simulations using each of the subsamples’ estimated parameters, our results suggest that the callo de hacha fishery is a profitable (positive NPV) activity in two out of four economic scenarios (Fig 2). The results indicate net profitability is obtained for scenarios 1 and 2, while scenarios 3 and 4 will lead to net losses.

Despite having positive NPV, in terms of distribution, scenarios 1 and 2 also present a high degree of dispersion. For both of them, the maximum NPV possible is more than 2 times the mean, while the difference for scenarios 3 and 4 is relatively small. In addition, the standard deviation for scenarios 1 and 2 is larger in proportion to the mean, than for scenarios 3 and 4 (Table 4). This result suggests that the most optimistic scenarios, despite predicting positive NPVs, also have a greater degree of uncertainty associated with their profitability.

**Table 4:** Summary of economic results for all scenarios.

	Min	Mean	Max	SD	95% Low	Median	95% High
**Scenario 1**	$ 3	$ 274	$ 565	$ 82	$ 138	$ 274	$ 409
**Scenario 2**	$ 27	$ 212	$ 455	$ 76	$ 86	$ 216	$ 337
**Scenario 3**	-$ 174	-$ 147	-$ 120	$ 8	-$ 160	-$ 147	-$ 134
**Scenario 4**	-$ 180	-$ 162	-$ 144	$ 6	-$ 172	-$ 163	-$ 153

Net Present Value is presented in thousands of US dollars ($).

**Fig 3:**
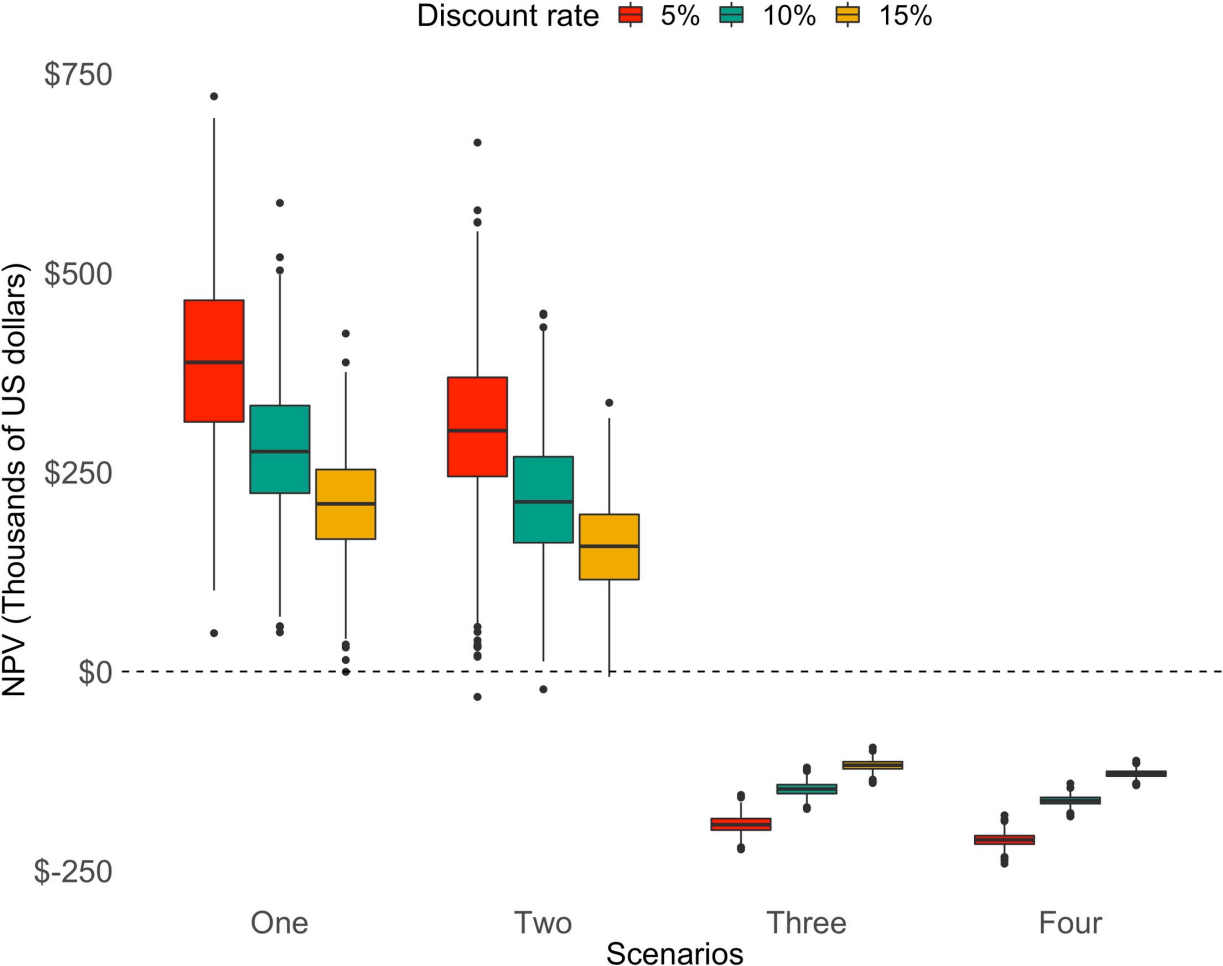
Net present value simulations for each *callo de hacha* scenario under different discount rates.

Studying the sensitivity of the results to the discount rate also rendered important insights (Fig 3). While scenarios 1 and 2 were able to remain positive on average, even with relatively high discount rates, scenarios 3 and 4 paint a dire situation for the fishery: the fishery is not profitable for even a single year. In a profitable fishery, discounting decreases the magnitude of positive long-run profits. This is the pattern observed for scenarios 1 and 2, where the fishery’s net present value decreases toward zero as the discount rate increases. When fishing activities are not able to cover monitoring and enforcement cost, the effect reverses. As discounting decreases, the magnitude of any payments or expenses in the long-run, every year losses of an unprofitable fishery become less negative, and negative present values will increase toward zero as the discount rate increases. This is the case for scenarios 3 and 4. In other words, in the less optimistic estimations the fishery is not even able to make up for the initial investment associated with monitoring, and the respective enforcement costs.

## Discussion

After four years of closure, the recovery efforts of NOS and the fishing community of *El Manglito* led to an overall increase in biomass and abundance of the *callo de hacha* species in the *Ensenada* of La Paz. The results in this study show that harvestable biomass, based on the most conservative scenario, showed a 260 percent growth in a four-year window. This sort of recovery is surprisingly in line with what has been seen in other scallop recovery efforts, such as the Atlantic sea scallop (*Placopecten magellanicus*) [30]. Nonetheless, there is no data available from before the fishing closure of the cove, making it impossible to establish where the estimated population stands in comparison to the population before the collapse. As a result of this effort, Mexican authorities re-opened the fishery in October of 2017 allowing 3.1 tons of callo to be harvest from the cove in that year, and a subsequent 7 tons in 2018 [31]. This level of production could only be achieved under our samples (and thus, scenarios) 1 and 2, although high uncertainty exists.

Population recovery is not necessarily surprising. If a fishery is declared under moratorium, then the population is expected to recover over time. This recovery, however, is a function of how effective the measure is at stopping fishing. What makes this case a valuable example for fisheries management is that the closure in the cove, in a context of absent management and poorly defined property rights, effectively led to a recovery of this fishery. We conjecture that this result was highly conditioned by community involvement.

The data allow us to visualize some of these dynamics at play. For example, initial years of low population levels could be attributed to the persistence of fishing activities. After the poorly-enforced closure of the fishery in 2008 [15,16], and because there was still an active market for the product, the momentum from open access limited the recovery effort. As community involvement started to take place, fishers got involved in monitoring activities and may have started to develop a sense of value to recovering the fishery. As time went by, this perceived value spread through the community, which increased compliance and rendered the moratorium effective.

It is possible that biological and environmental conditions also contributed to this pattern. For example, as these scallops tend to aggregate, it wasn’t until sufficient density was accumulated that recruitment was able to increase [32]. As this mechanism unfolds over time and in space, it is only when enough individuals are concentrated in reduced patches that biomass is able to take off. The effectiveness of this process, however, is still dependent on fishers respecting the moratorium. Hence, our intuition regarding the importance of community involvement in the entire process is confirmed.

In this paper, we have used data gathered by the fishing community to document the effectiveness of the recovery effort, but our analysis it not without caveats. One important issue to highlight is the ample differences in biomass estimations between samples 1 and 2, and samples 3 and 4 (Table 2). The samples differ in the inclusion of the 16 quadrats that presented high abundance. When we asked stakeholders directly about these locations, fishers identified these quadrats as areas with historically high density patches where the seafloor exhibits irregularities. Such irregularities have been recognized as natural mechanisms for larvae sink and could potentially explain those differences in biomass [33]. An additional environmental feature of the system is the division between the in/out flow of the inner Gulf of California, which creates a heterogeneous environment for the resource [33]. It is possible that the high abundance seen in samples 1 and 2 are a result of a combination between these macro- and micro-scale environmental processes. Future efforts for a sound management of the cove, and other areas where *callo de hacha* is extracted, will benefit from taking these ocean dynamics into account.

Given our research design, these disparities in biomass estimation directly permeated the economic scenarios. According to our model, if *callo de hacha* was to be harvested and properly managed at the time of the present study, it would only be profitable under scenarios 1 and 2 (Fig 2). We estimate that the fishery could only provide employment for 104 (Scenario 1) and 81 (Scenario 2) fishers with a minimum wage per fishing season [34], respectively. It is important to note, however, that these levels of profits reflect a fixed individual-muscle (edible part) ratio, and profits may grow larger if fishers target animals with bigger adductor muscles. Management strategies taking economic considerations into account would also benefit from taking this assumption into consideration.

For scenarios 3 and 4, which do not consider the high-density quadrats, our results suggest that the fishery may not be profitable. The main reason for this result is the low harvestable biomass that these scenarios provide and the high fixed cost of enforcing the area. Removing this cost makes scenarios 3 and 4 profitable with an average NPV of US$ 55 and 40 thousand, respectively (S2 Fig). The high sensitivity of the economic results is an important result of this paper. Enforcing illegal fishing activities is a responsibility of the Mexican government by law [11]. Nevertheless, management problems, scarcity of public funds, and a lack of infrastructure have greatly reduced the ability of the authorities to conduct these duties [13,14]. As the community is forced to undertake enforcement duties on its own, the value of the resource is effectively reduced, and the incentives to follow sound management practices start to disappear. It is only natural to suggest that this line of incentives is what led the fishery to a collapse in the first place.

Acknowledging this reality is crucial for the success of any recovery effort. In the case of the *callo de hacha*, external funds have helped to cover the recovery effort in the community, but long-term success is highly dependent on maintaining the profitability of the activity and incentives aligned across the community. If the recovered stock indeed falls into our most pessimistic scenarios, either proper government enforcement or externally provided funds will be key to maintaining community involvement in sustainable practices. Otherwise, the potential value of the fishery starts to dissipate and fishers will lose any incentive to maintain the population at sustainable levels. How self-sufficient these recoveries efforts are should be one of the most important discussions when it comes to design and implementation of recovery plans. This requirement is especially true when discussing the potential benefits with the fishers involved.

Having an open discussion about benefits and costs associated with the recovery of a fishery, however, also requires a good information set upon which to build that discussion. Unfortunately, many of the fisheries that find themselves in trouble often lack the required data associated with this process [35]. Our case is not different. While this analysis was only made possible by data gathered by the fishers themselves, there is much that can be improved. Future monitoring efforts in the *Ensenada*, and other similar fisheries, will benefit from reducing variation within the sampled data. Some approaches include standardizing methods and maintaining them throughout time. Efforts in the cove are headed in that direction. Spawning data started to be collected in 2014, which will allow better assessments of population dynamics (e.g., is the population self-maintained?; S1 Fig). Improving these aspects will certainly reduce uncertainty in the biomass estimation and in any future economic analyses.

The biomass increase in the cove and the potential profitability of the fishery highlights several components that can inform future recovery efforts in other small-scale fisheries. Reducing fishing mortality to zero is not always straightforward. Often, fishers are highly dependent on extraction and, following their own personal and valid needs, they are eventually forced to keep fishing during closures [21,36]. For our case study, we have suggested that investing in community involvement was not only fundamental to ensure compliance, but it also allowed the monitoring of the recovery process [22,37,38]. In addition, the effort of more than four years of restoration plans also resulted in the *Organización de Pescadores Rescatando la Ensenada*. By adopting this single cooperative structure, fishers were able to request an exclusive concession for the resource, shifting the management status from de facto open access to a property-right based system [12]. This transition creates incentives for maximizing and maintaining the value of the resource, which is expected to encourage sustainable practices in the long run.

Having evidence of the biological restoration and profitability of the fishery, the Mexican authorities re-opened the fishery in 2017 after their own evaluation. The fishers’ organization was consigned a total of 3.1 tons of *callo* allocated to be harvested in the cove [31]. This experience has provided many learning points to the fishers in the *Ensenada*, including how to comply with closures, how to monitor the status of the resource, and how to organize and secure property rights over a resource. More importantly, these experiences also shed light on potential approaches for other artisanal communities in Mexico and the rest of the world.

## Conclusion

After more than four years of restoration management, the fisher community of *El Manglit*o started cooperating and together managed successfully to recover the *callo de hacha* fishery. The involvement of the NGO *Noroeste Sustentable* with the community supported not only the recovering of natural capital, but also created momentum to build social and cultural capital. The combination of these factors has allowed the community a new opportunity to derive benefits from the resource in a sustainable manner. While these results are promising, there is still work that can be done in the sampling and management stages to reduce uncertainty. Taking in consideration the high economic sensitivity of this fishery to enforcement costs will help the community make the most of the recently re-opened fishery. Needless to say, an increment in the magnitude seen in this fishery is definitely a sign of improvement. The restoration of the *callo de hacha* population in the *Ensenada* of La Paz proved to be a successful example of fisheries restoration based on a bio-social approach that could be adopted by similar small scale fishing communities in Latin America and the world.

## Supporting information

S1 Data Biological data used for the current analysis. (ZIP)

S1 Fig Relation between recruits and spawning biomass to project the population. (PNG)

S2 Fig Net present value simulations for each *callo de hacha* scenario without enforcement costs. (PNG)

S1 File Abstract in Spanish (*Resumen en español*). (DOCX)

S2 File Economic model tool. (ZIP)

S1 Table Biological assumptions of the model. (DOCX)

S2 Table Description of the biological factors and economic inputs used to develop the economic model. (DOCX)
