# Supplementary figures and images for: Evaluating the bio-economic performance of a Callo de hacha (Atrina maura, Atrina tuberculosa & Pinna rugosa) fishery restoration plan in La Paz, Mexico
Source: PLoS One. 2018 Dec 20;13(12):e0209431. doi: 10.1371/journal.pone.0209431 (PMC6301776; doi:10.1371/journal.pone.0209431)

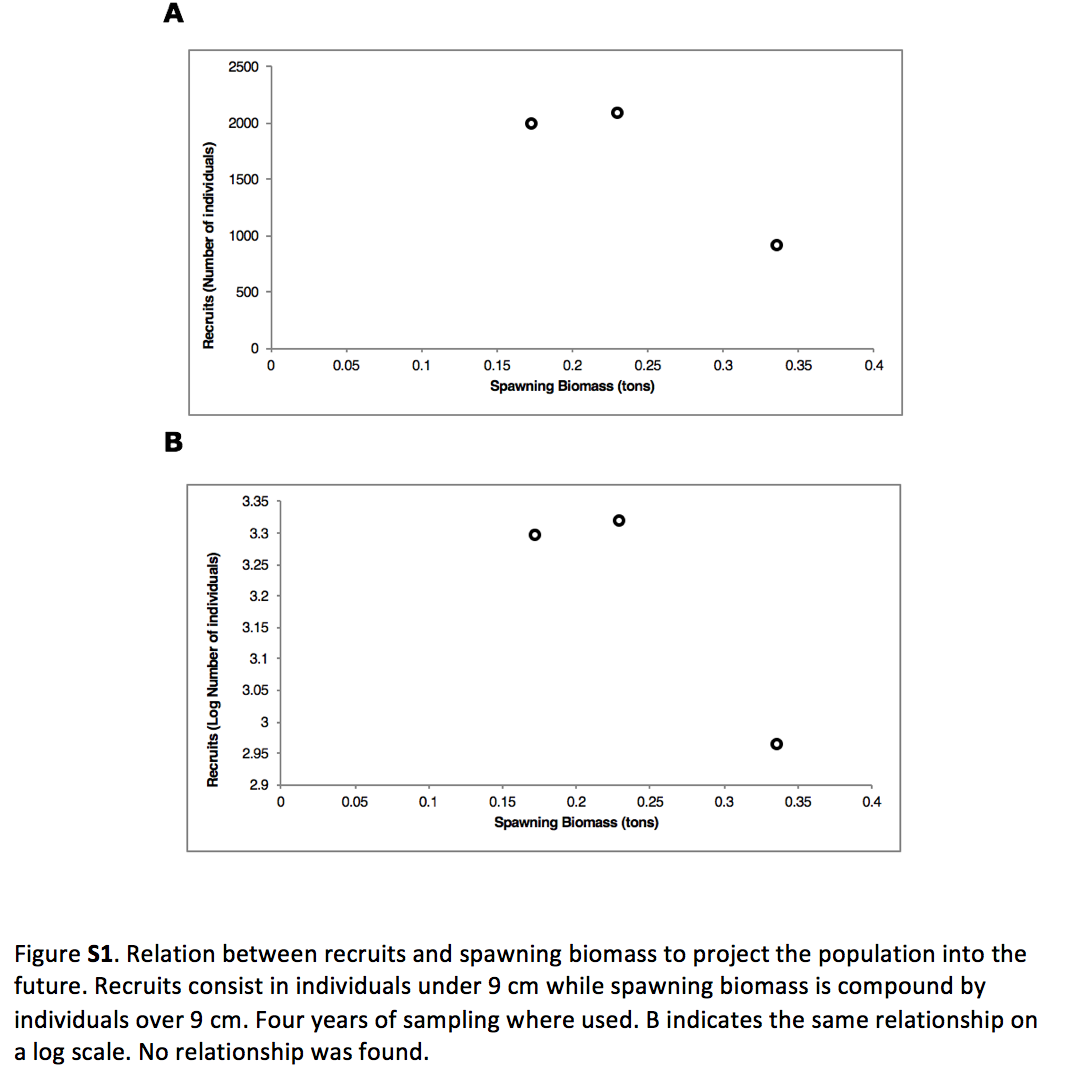

Supplement: S1 Fig — (PNG) [file pone.0209431.s002.png]

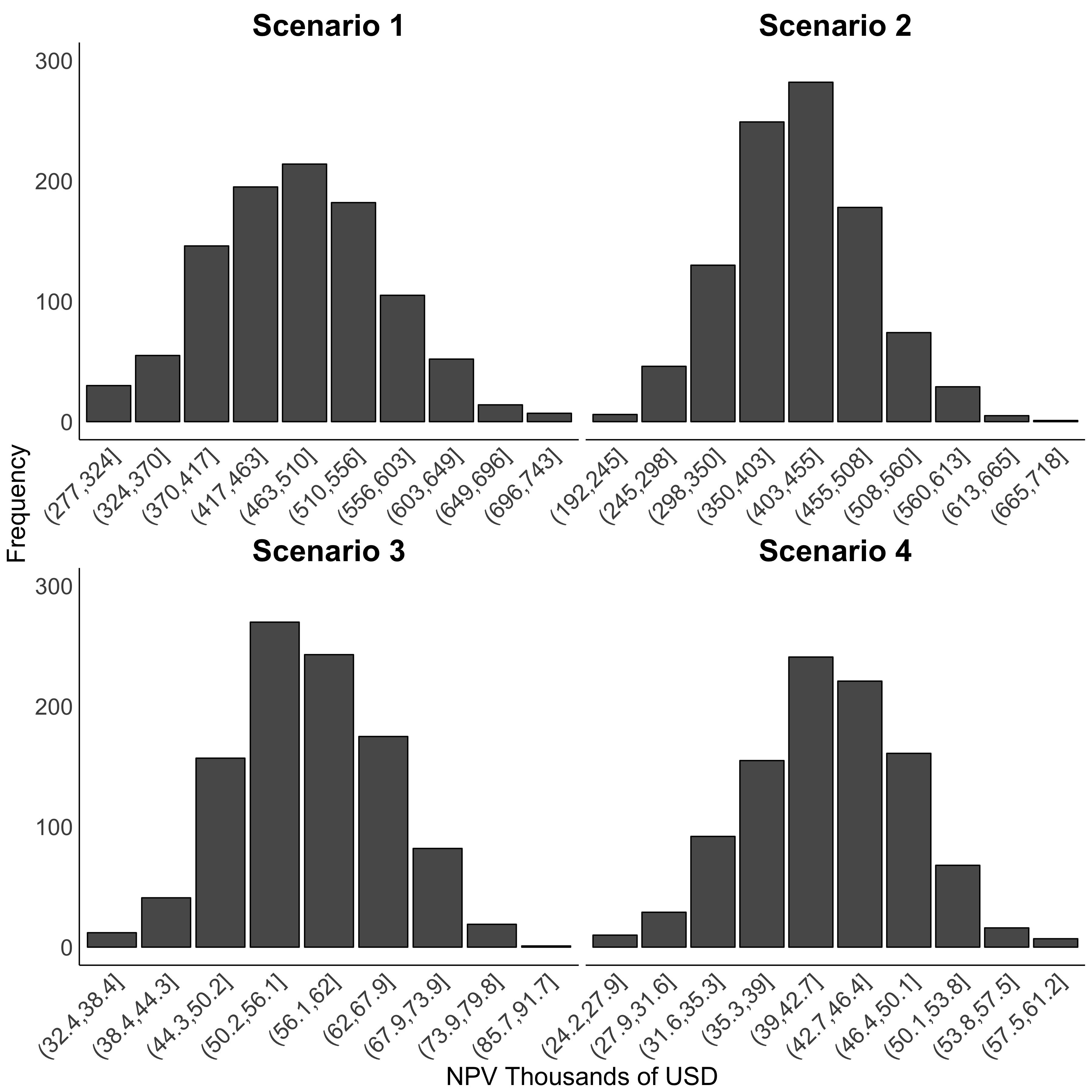

Supplement: S2 Fig — (PNG) [file pone.0209431.s003.png]
