# Supplementary material for: Evaluating the bio-economic performance of a Callo de hacha (Atrina maura, Atrina tuberculosa & Pinna rugosa) fishery restoration plan in La Paz, Mexico
Source: PLoS One. 2018 Dec 20;13(12):e0209431. doi: 10.1371/journal.pone.0209431 (PMC6301776; doi:10.1371/journal.pone.0209431)
Supplement: S1 File — (DOCX) [file pone.0209431.s004.docx]

**Título:** Evaluación bio-económica de la restauración de la pesquería de *Callo de hacha (Atrina maura, Atrina tuberculosa & Pinna rugosa)* en la bahía de La Paz, Baja California Sur, México.

**Autores:** Juliano Palacios-Abrantes, Juliana Herrera-Correal, Salvador Rodriguez, Jacy Brunkow, Renato Molina.

**Resumen:**

La pesca artesanal contribuye en gran medida a las economías regionales y los medios de subsistencia en las comunidades costeras de América Latina. Si bien México es uno de los casos en que la pesca artesanal desempeña un papel importante, la sobrepesca y estrategias de manejo mal implementadas han llevado varias pesquerías al colapso. Un ejemplo es la pesquería de callo de hacha de la ensenada de La Paz en Baja California Sur, que, después de años de mala gestión, fue cerrada por las autoridades mexicanas en 2009. El presente estudio evaluó los esfuerzos de recuperación de dicha pesquería en la ensenada de La Paz, resultado de una colaboración entre una organización no gubernamental y la comunidad pesquera. Después de cuatro años de cierre y monitoreo activo del proceso de recuperación, la población de callo de hacha ha mostrado una recuperación de la población estadísticamente significativa, con potencial de solvencia para reabrir las actividades pesqueras. Se evaluaron cuatro escenarios de incertidumbre de los cuales dos proporcionan valores actuales netos positivos. También se documentan aspectos clave de la relación entre la organización no gubernamental y la comunidad pesquera, que crearon capital cultural y social y, en nuestra opinión, fue esencial para una restauración exitosa. Tener una comunidad activamente involucrada ayudó a recaudar fondos para el cierre de pesca y para que los pescadores pudieran cumplir con la legislación mexicana, también fomentó la construcción comunitaria y la auto organización, que fueron un punto clave en la recuperación del recurso y serán cruciales para mantener la sostenibilidad de la industria pesquera.

**Palabras clave:** restauración pesquera, callo de hacha, ostiones, pesquerías artesanal
