# Supplementary material for: Evaluating the bio-economic performance of a Callo de hacha (Atrina maura, Atrina tuberculosa & Pinna rugosa) fishery restoration plan in La Paz, Mexico
Source: PLoS One. 2018 Dec 20;13(12):e0209431. doi: 10.1371/journal.pone.0209431 (PMC6301776; doi:10.1371/journal.pone.0209431)
Supplement: S1 Table — (DOCX) [file pone.0209431.s006.docx]

**S1 Table. Biological assumptions of the model**

| **Assumptions** | **Justification** |
| --- | --- |
| All species are treated as one | Data set for *P. rugosa* and *A. tuberculosa* was not enough to do a species level analysis. Moreover, literature review on the biological model inputs did not prove important differences within species (Basurto, 2008). |
| There is no spatial difference within the habitable area | The actual sampling method does not allow doing a spatial comparison within the habitable resource habitat. |
| Fishing mortality (2011-2014) equals 0 | According to NOS the community stopped fishing since 2011. The model does not account for any potential illegal fishing thought those years. |
| Zig-Zag method covers all quadrat area | According to NOS, the zig-zag method covered all area of the quadrat. |
| Aggregation was not accounted for | These animals tend to aggregate, however the data set limits the information needed to develop a model that could consider potential individual aggregation. |
| The Ensenada has two different populations of *callo de hacha* | It was assumed that there were two populations without any interaction in order to aggregate the biomass of “normal” quadrats and “highly abundant ones”. |
| Individual – muscle conversion ratio | It is assumed that muscle mass represents 25% of total individual weight (Camacho-Mondragon et al., 2012) |

Basurto, X. (2008). Biological and ecological mechanisms supporting marine self-

governance: The Seri Callo de Hacha fishery. *Ecology and Society*.

Camacho-Mondragón, M. A., Arellano-Martínez, M., & Ceballos-Vázquez, B. P. (2012).

Particular features of gonadal maturation and size at first maturity in *Atrina maura* (Bivalvia: Pinnidae). *Scientia Marina*, *76*(3), 539–548. http://doi.org/10.3989/scimar.03522.05A
