# Supplementary material for: Evaluating the bio-economic performance of a Callo de hacha (Atrina maura, Atrina tuberculosa & Pinna rugosa) fishery restoration plan in La Paz, Mexico
Source: PLoS One. 2018 Dec 20;13(12):e0209431. doi: 10.1371/journal.pone.0209431 (PMC6301776; doi:10.1371/journal.pone.0209431)
Supplement: S2 Table — (DOCX) [file pone.0209431.s007.docx]

**S2 Table.** Description of the biological factors and economic inputs used to develop the economic model.

| **Input** | **Variable** | **Definition** | |
| --- | --- | --- | --- |
| **Biological** | Mean | Harvestable biomass mean of that years sampling | |
|  | Standard deviation | Statistical Standard deviation of the harvestable biomass mean | |
|  | Fishing mortality | Fish mortality due to fishing | |
|  | Conversion factor | Individual to muscle (*callo)*  rate | |
| **Economic** | Price Per Kilo | Selling price of the product (fisherman) | |
|  | Fishing Cost | Operational and administrative costs that are spend per year |  |
|  | Fix Cost | Cost of fishing, answers the question: how much does a fishing trip costs? | |
|  | Cost per unit of effort | Cost of each fishing trip, value is related to the total amount of fishing trips and the CPUE | |
|  | Cost per unit of catch | Cost of processing the product, value is linked to total capture | |
|  | Discount rate | Future value of present money | |
|  | Depreciation | Years of depreciation |  |
|  | Tax | Selling TAX over product (Estimated at 15%) |  |
|  | Inversion | Any initial inversion fisherman must make |  |
|  | Other Sources of income | Any other source of income | |
